# Caffeine citrate enhanced cisplatin antitumor effects in osteosarcoma and fibrosarcoma in vitro and in vivo

**DOI:** 10.1186/s12885-019-5891-y

**Published:** 2019-07-15

**Authors:** Kensaku Abe, Norio Yamamoto, Katsuhiro Hayashi, Akihiko Takeuchi, Hiroyuki Tsuchiya

**Affiliations:** Department of Orthopaedic Surgery, Graduate School of Medical Sciences, Kanazawa University, 13-1 Takara-machi, Kanazawa, 920-8641 Japan

**Keywords:** Caffeine citrate, Chemotherapy, Osteosarcoma, Fibrosarcoma, Cancer

## Abstract

**Background:**

While multiagent chemotherapy has dramatically improved the prognosis of sarcoma, the novel chemotherapeutics have hardly developed over the past 30 years. Caffeine can induce apoptosis, delays in cell cycle progression and can enhance the cytocidal effects of anti-cancer agents. Citrate has been reported to enhance the cytocidal effect of cisplatin in gastric cancer in vitro. However its effect in sarcoma cells had not been reported.

**Methods:**

This study was designed to evaluate whether the addition of caffeine, citrate, or caffeine citrate to cisplatin improved its cytocidal effect (cell survival, proliferation, and apoptosis) on human osteosarcoma (HOS), human fibrosarcoma (HT1080) and murine osteosarcoma (LM8) cell lines. We also tested the various combinations in a mouse heterotopic transplantation model in vivo*.* In cell survival assay, combination index (CI) of caffeine citrate was calculated as a combination of anhydrous caffeine and citric acid, and the synergy was evaluated (CI < 1.0).

**Results:**

In all cell lines, cisplatin combined with caffeine citrate significantly reinforced the anticancer effect compared with cisplatin alone, combination of cisplatin and anhydrous caffeine, and combination of cisplatin and citric acid. Moreover, CI was < 1.0 in all conditions. The anticancer agent reinforcement effect of caffeine citrate was synergy of anhydrous caffeine and citric acid. In cell proliferation and cell cycle assay revealed that caffeine citrate had most strong effect as a combination drug than caffeine and citric acid in inducing G0/G1 cell-cycle arrest with subsequent suppressed cell proliferation. In mitochondrial depolarization and caspase 3/7 activity assay revealed that caffeine citrate had most strong effect as a combination drug than caffeine and citric acid in apoptosis associated with decreased mitochondrial membrane potential. In vivo, three different drug concentrations were tested, and cisplatin combined with caffeine citrate was found to have the strongest antitumor effect.

**Conclusions:**

This is the first report demonstrating that caffeine citrate has a significantly greater potentiating effect on cisplatin than adding either caffeine or citric acid. The combination of cisplatin with caffeine citrate is a novel treatment that might hold promise for improving the outcome of osteosarcoma and fibrosarcoma, which up till now has generally not responded well to chemotherapy.

**Electronic supplementary material:**

The online version of this article (10.1186/s12885-019-5891-y) contains supplementary material, which is available to authorized users.

## Background

Osteosarcoma is a malignant primary bone tumor occurring mainly in adolescents and young adults [1]. The 5-year survival is approximately 70% [2–4]. However, patients with metastatic disease at diagnosis or with recurrent disease have a 5-year survival of only 20% [5]. Metastases most commonly affect the lung and are the most common cause of death in patients with osteosarcoma [6]. First-line therapy for this malignancy consists of high-dose methotrexate, cisplatin, doxorubicin, and ifosfamide. However, dose-intensive chemotherapy regimens with these agents have not improved outcomes [7].

Soft tissue sarcoma can arise in almost any anatomic site, including the extremities (60% of cases), thorax, abdomen, retroperitoneum, or head and neck [8]. More than 50 histologic subtypes of soft tissue sarcoma have been identified and are defined by the World Health Organization classification, each with a different treatment response and prognosis [9, 10]. The 5-year survival of patients with the entire range of soft tissue sarcoma remains about 50%, which is far from satisfactory [8, 11]. In advanced soft tissue sarcoma that is either unresectable or metastatic, the 5-year survival reportedly ranges from 20 to 50% [8]. Drugs used in first-line treatment include doxorubicin, ifosfamide, and dacarbazine, either alone or in combination [12–17]. New drugs (pazopanib, trabectedin, and eriblin) have been introduced, but it has not been effective enough to become first-line chemotherapy instead of existing anticancer drugs.

Caffeine (1,3,7-trimethylxanthine) [18] has been shown to induce apoptosis [19–22], overcome chemotherapy- or radiation-induced delays in cell cycle progression [23], and enhance the toxicity of radiation and anticancer agents [24]. In 1989, we developed caffeine-potentiated chemotherapy for malignant bone and soft tissue tumors and subsequently demonstrated the clinical effectiveness of this regimen in clinical use [25–27]. Citrate has been reported to have the ability of the potentiation of anticancer drugs, induction of apoptosis in several cancers other than sarcoma in vitro [28–32]. We designed this study to investigate whether the combination of cisplatin and caffeine citrate show the antitumor effect in sarcoma cell lines in vitro and in vivo.

## Methods

### Drugs

Cisplatin (Wako Pure Chemical Industries, Ltd., Osaka, Japan), caffeine (Wako Pure Chemical Industries, Ltd., Osaka, Japan), and citrate (Wako Pure Chemical Industries, Ltd., Osaka, Japan) and caffeine citrate (Respia, Nobelpharma Co., Ltd. Tokyo, Japan) were used.

### **Cell lines and culture**

Human osteosarcoma (HOS), human fibrosarcoma (HT1080), and mouse osteosarcoma (LM8) cell lines (American type culture collection, Manassas, VA, USA; Takara bio Inc., Japan) were used in the experiments. All cells were grown in RPMI 1640 medium (Gibco, Grand Island, NY, USA) supplemented with 10% fetal bovine serum, 100 U/ml penicillin, and 100 μg/ml streptomycin.

### **Cell survival assay**

Cell viability was assessed using the WST-8 (4-[3-(4-iodophenyl)-2-(4-nitrophenyl)-2H-5-tetrazolio]-1,3-benzene disulfonate) assay kit (cell counting Kit-8, Dojindo laboratory, Mashikimachi, Japan). Briefly, cells were seeded in 96-well flat-bottomed microplates (5 × 10^3^ cells/well), incubated at 37 °C for 24 h, and exposed to various concentrations of cisplatin (0, 0.125, 0.25, 0.5, 1.0, and 2.0 μM) alone or with the addition of 0.5 mM of caffeine, citrate, or caffeine citrate. Total amount of medium was 200 μl/well. At least 4 wells were used for each of the concentrations tested. After incubation with the test compounds for 72 h, 10 μl WST-8 solution was added to each well. The microplates were incubated for another 3 h at 37 °C, and absorption at 450 nm was measured using a microprocessor-controlled microplate reader (iMark microplate absorbance reader, bio-rad laboratories, Inc., Hercules, CA). The cell-survival fraction was calculated as the percentage of untreated control cells, and the half-maximal inhibitory concentration (IC_50_) values were derived.

### **Calculation of combination index**

A combination index (CI) assay was performed to test whether the addition of caffeine, citrate, or caffeine citrate enhanced the antitumor effect of cisplatin in the HOS, HT1080, or LM8 cell lines using the CalcuSyn software from ComboSyn Inc. (New Jersey, USA) [33]. CI was calculated by median effect and isobologram methods [34–37]. Synergy was defined as CI < 1.0, antagonism as CI > 1.0, and additive effect at CI values not significantly different from 1.0.

### **Cell proliferation assay**

We evaluated cell proliferation following treatment with 0 μM or 0.25 μM of cisplatin alone or combined with 0.5 mM of caffeine, citrate, or caffeine citrate for 24 h, using a click-iT plus EdU Alexa Fluor 555 imaging kit (Fluoroskan ascent FL; Labsystems, Thermo fisher scientific, Waltham, MA, USA; EdU standing for 5-ethynyl-2-deoxyuridine). Briefly, HOS, HT1080, and LM8 cells were seeded on slide chambers and incubated overnight. After treatment, cells were treated with EdU (10 mmol/L) for 1 h. then, they were fixed with 4% paraformaldehyde (Wako pure chemical industries, ltd., Osaka, Japan), washed with 3% bovine serum albumin in PBS, and permeabilized with 0.5% triton X-100. Cells were then incubated with the click-iT reaction cocktail, followed by Hoechst 33342 (NucBlue live ReadyProbes reagent, Invitrogen, Carlsbad, CA), and were observed using fluorescence microscope (BZ-9000, Keyence co., Osaka, Japan). EdU-positive cells were scored for cells treated with 0 μM of cisplatin or 0.25 μM of cisplatin with or without the other compounds. In this assay, results were reported as the mean percentage of EdU-positive cells in five microscopic fields ± standard deviation (SD).

### **Mitochondrial depolarization assay**

We evaluated the mitochondrial membrane potential following treatment with 0 μM of or 0.25 μM of cisplatin alone or combined with 0.5 mM of caffeine, citrate, or caffeine citrate for 24 h to detect mitochondrial membrane potential. Briefly, HOS, HT1080, and LM8 cells were seeded on slide chambers and incubated overnight. After treatment, unfixed live cells were stained by incubating them with 100 nM tetramethylrhodamine methyl ester (TMRE) (ab113852, Abcam plc, Cambridge, UK) in the dark for 30 min at 37 °C in 5% CO_2_, followed by Hoechst 33342 (NucBlue live ReadyProbes reagent, Invitrogen, Carlsbad, CA). The cells were washed with PBS and then examined under a fluorescence microscope (BZ-9000, Keyence CO., Osaka, Japan). The results were reported as the mean luminance of one cell in five microscopic fields ± SD. The luminance was calculated by the analysis application (BZ-II analyzer Ver. 1.42,Keyence co., Osaka, Japan).

### Flow cytometry

#### **Cell cycle assay**

We evaluated cell cycle profile following treatment with 0 μM or 0.25 μM of cisplatin alone or combined with 0.5 mM of caffeine, citrate, or caffeine citrate for 72 h, using the MUSE cell cycle kit with the MUSE cell analyzer (Merck Millipore, Billerica, MA, USA) according to the manufacturer’s protocol. Briefly, 72 h after treatment, HOS cell, as a representative cell, was used. Around 1 × 10^6^ cells were transferred to a 2 ml tube. The cells were centrifuged at 300×g for 5 min. The cell pellets were washed twice with PBS. The washed cells were fixed with 70% ethanol. For fixation, cells were incubated for 3 h at − 20 °C. about 200 μl of fixed cells and an equal volume of Muse cell cycle reagent were mixed and incubated for 30 min at room temperature in dark. Then, cell cycle was analyzed using the kit described above.

#### **Caspase 3/7 activity assay**

Caspase-3/7 activity was evaluated following treatment with 0 μM or 0.25 μM of cisplatin alone or combined with 0.5 mM of caffeine, citrate, or caffeine citrate for 72 h, using the MUSE Caspase-3/7 kit with the MUSE cell analyzer (Merck Millipore, Billerica, MA, USA) according to the manufacturer’s protocol. Briefly, 72 h after treatment, HOS cell, as a representative cell, was used. Cell samples were incubated with muse Caspase-3/7 working solution in the dark for 30 min at 37 degrees. Next, the muse 7-AAD working solution was added for 5 min. Samples were read on the muse cell analyzer and results were reported as percentages of live (lower left quadrant), apoptotic (lower right quadrant), apoptotic/dead (upper right quadrant) and dead (upper left quadrant) cells.

### **In vivo tumorigenesis assay**

**Fig. 1:**
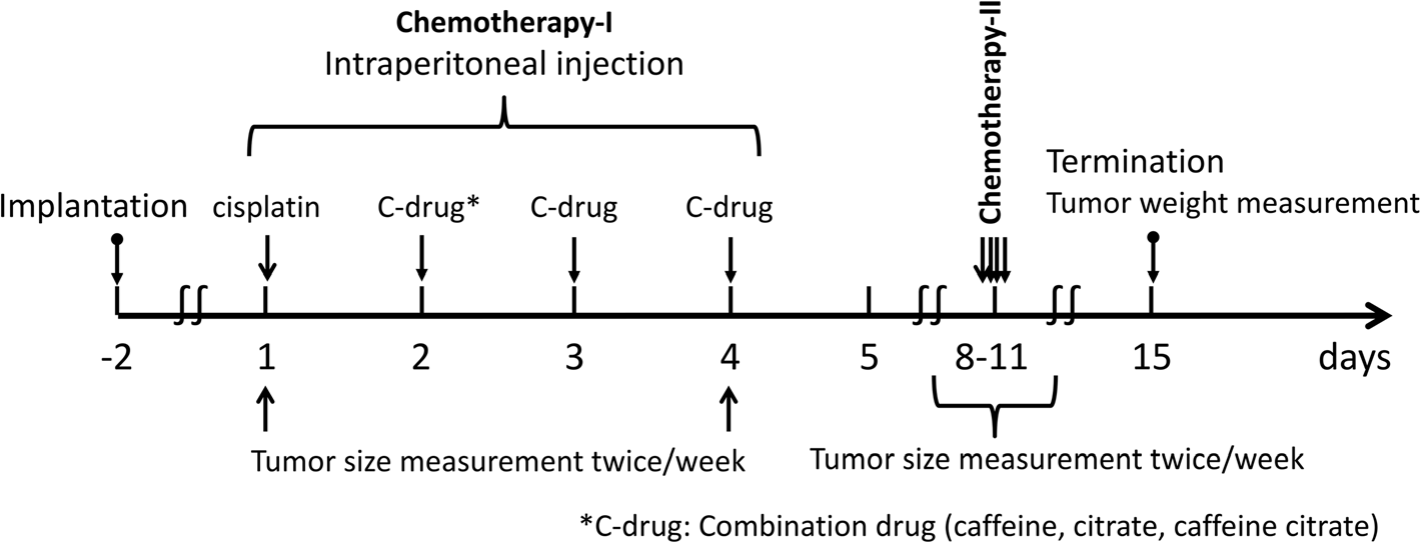
Treatment scheme. 2 days after tumor cells were injected, chemotherapy was begun. The tumors were measured on days 1 and 4 of each cycle. C-drug, Combination drug: caffeine, citrate, or caffeine citrate

4-week-old female athymic nude mice (Charles River laboratories Japan, INC) were used for the tumorigenesis. LM8 and HT1080 cells were suspended (5 × 10^5^ cells/100 μl) in Matrigel (BD bioscience, New Jersey, USA) and injected subcutaneously with a 1.0 ml 27-G latex-free insulin syringe (Terumo medical corporation, Japan). The mice were assigned to groups (3–4 mice each) and given cisplatin with or without caffeine, citrate, or caffeine citrate with intraperitoneal injections. Physiologic saline was used as a control. A treatment course was given over 1 week, with cisplatin (or saline) administered on day 1 and caffeine, citrate, or caffeine citrate administered on days 2 to 4, followed by a drug holiday on days 5 to 7, and we repeated two courses of the treatment with same time course (Fig. 1). We examined three treatment protocols according to our previous study [38, 39]. The regimen 1(R1) was composed of cisplatin (6 mg/kg body weight), caffeine (100 mg/kg), citrate (100 mg/kg), and caffeine citrate (200 mg/kg). The regimen 2 (R2) was composed of cisplatin (6 mg/kg body weight), caffeine (50 mg/kg), citrate (50 mg/kg), and caffeine citrate (100 mg/kg). The regimen 3 (R3) was composed of cisplatin (3 mg/kg body weight), caffeine (100 mg/kg), citrate (100 mg/kg), and caffeine citrate (200 mg/kg). The R2 and R3 were administrated for osteosarcoma cells. Throughout the experiment, all mice were carefully observed daily for adverse events. Another set of mice without implanted tumors were treated with three courses of the R1 to measure changes in body weight. Tumor volume was measured in two dimensions twice a week on days 1 and 4. Tumor volume (cm^3^) was calculated using the formula: 0.5 × *a*^2^ × *b*, where *a* was the smallest tumor diameter (cm) and *b* the largest [40]. After two courses of chemotherapy, the mice were euthanized by large amount intraperitoneal injection of pentobarbital sodium and the tumors removed and weighed.

All animal experiments were undertaken in accordance with the Guidelines for the Care and Use of Laboratory Animals under the National Institutes of Health assurance number A3873–01 and the U.S. Public Health Service Policy on Humane Care and Use of Laboratory Animals [41], which correspond to national guidelines in Japan [42]. The humane endpoint of the maximum tumor size was set which was not exceed 10% of normal body weight according (Institutional Animal Care and Use Committee) guidelines [43]. The protocol was approved by the Institute for Experimental Animals, Kanazawa University Advanced Science Research Center.

### **Statistical analysis**

The data were statistically compared by the ANOVA using Statcel 3 (the Publisher OMS ltd., Tokyo, Japan). A *p* value of < 0.05 was considered statistically significant.

## Results

### **Effect of cisplatin and combinations on cell survival and combination index**

**Fig. 2:**
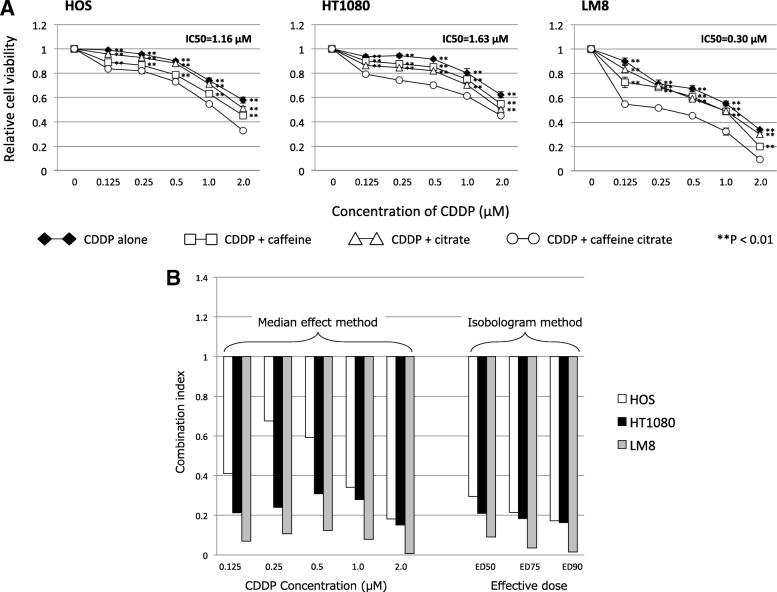
(**a**) Effect of caffeine citrate-potentiated chemotherapy on the survival of human (HOS) and mouse (LM8) osteosarcoma cells and human fibrosarcoma cells (HT1080). The cells were treated for 72 h with physiologic saline as a control or with the indicated concentration of cisplatin (CDDP) alone or combined with caffeine, citrate, or caffeine citrate. Relative numbers of viable cells were measured by the WST-8 assay. Values shown are the means ± standard deviation of four separate experiments. (**b**) The combination index values for cisplatin (CDDP) + caffeine citrate were significantly < 1, indicating a synergistic effect at all CDDP doses in treating human (HOS) and mouse (LM8) osteosarcoma cells and human fibrosarcoma cells (HT1080)

We examined the effect of cisplatin combined with the other compounds on tumor cell survival. The viability of all cells was inhibited by cisplatin, cisplatin + caffeine, cisplatin + citrate, and cisplatin + caffeine citrate with a dose-dependent manner (Fig. 2a). IC_50_ values at 72 h after administration of cisplatin + caffeine citrate for HOS, HT1080, and LM8 cells were 1.16 μmol/l, 1.63 μmol/l, and 0.30 μmol/l, respectively. In both the median effect and isobologram methods, the CI value was less than 1.0, thereby demonstrating synergy between caffeine citrate and cisplatin at almost all tested concentrations in HOS, HT1080, LM8 cells (Fig. 2b).

### **Effect of cisplatin and combinations on cell proliferation**

**Fig. 3:**
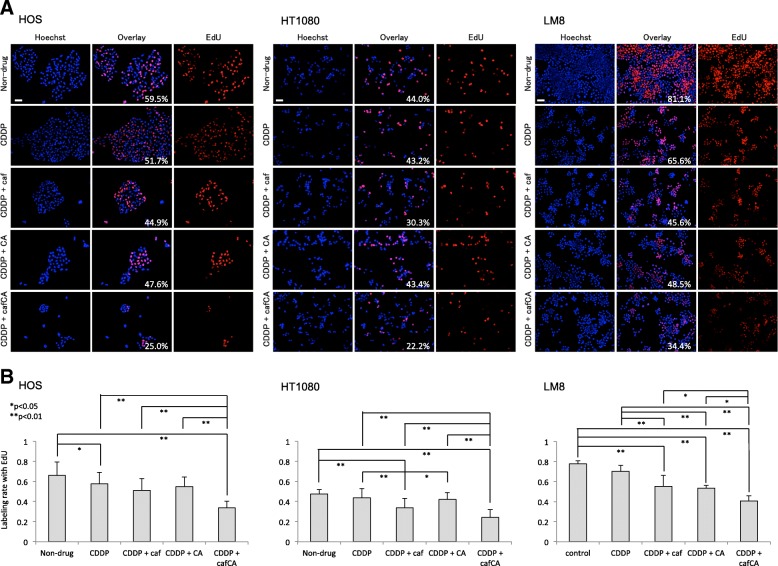
Effects of cisplatin (CDDP) alone or with caffeine (caf), citrate (CA), or caffeine citrate (cafCA) on proliferation of human (HOS) and human fibrosarcoma cells (HT1080). **a** Representative immunofluorescence microscopic findings of expression of the EdU-positive proliferating cells, including overlay with Hoechst-stained micrographs. Scale bar = 50 μm. The number in the Overlay panels is the rate of EdU-positive cells. **b** Effects of the various treatments on the numbers of EdU-positive proliferating cells. Data are mean percentages of EdU-positive proliferating cells in five microscopic fields with standard deviations EdU, 5-ethynyl-2-deoxyuridine.

In EdU assay, cisplatin + caffeine citrate resulted in a significant decrease in EdU-positive proliferating cells in the HOS, HT1080, and LM8 cells (Fig. 3). In HOS cells, EdU-positive cells were significantly decreased in cisplatin + caffeine and cisplatin + caffeine citrate relative to the control group, and in cisplatin + caffeine citrate relative to all other treatment groups. In HT1080 cells, EdU-positive cells were significantly decreased in cisplatin + caffeine/caffeine citrate relative to the control group, in cisplatin + caffeine relative to cisplatin and cisplatin + citrate, and in cisplatin + caffeine citrate relative to all other treatment groups. In LM8 cells, EdU-positive cells were significantly decreased in cisplatin + caffeine/citrate/caffeine citrate relative to the control group and cisplatin, and in cisplatin + caffeine citrate relative to all other treatment groups.

### **Effect of cisplatin and combinations on mitochondrial membrane potential**

**Fig. 4:**
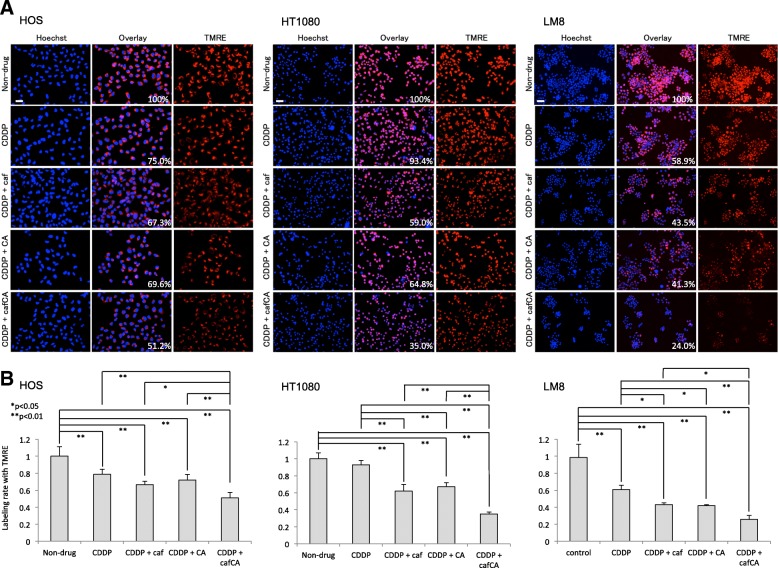
Effects of cisplatin (CDDP) alone or with caffeine (caf), citrate (CA), or caffeine citrate (cafCA) on apoptosis of human (HOS) and human fibrosarcoma cells (HT1080). **a** Representative immunofluorescence microscopic findings of mitochondrial membrane potential expression. Scale bar = 50 μm. The number in the Overlay panels shows the intensity of luminance per cell when the non-drug is based on 100%. **b** Effects of various treatments on the mean luminance of mitochondrial membrane potential. Data are mean luminance of mitochondrial membrane potential in five microscopic fields with standard deviations. TMRE, tetramethylrhodamine methyl ester

In mitochondrial depolarization assay, cisplatin + caffeine citrate significantly decreased cell luminance, indicating a weaker mitochondrial membrane potential in the HOS, HT1080, and LM8 cells (Fig. 4). In HOS cells, the cell luminance was significantly decreased in all treatment groups relative to the control group, and in cisplatin + caffeine citrate relative to all other treatment groups. In HT1080 cells, the cell luminance was significantly decreased in cisplatin + caffeine/citrate/caffeine citrate relative to the control group and cisplatin alone, and in cisplatin + caffeine citrate relative to cisplatin + caffeine/citrate. In LM8 cells, the cell luminance was significantly decreased in all treatment groups relative to the control group, in cisplatin + caffeine/citrate/caffeine + citrate relative to cisplatin alone, and cisplatin + caffeine citrate relative to isplatin + caffeine.

### **Effect of cisplatin and combinations on cell cycle profile**

**Fig. 5:**
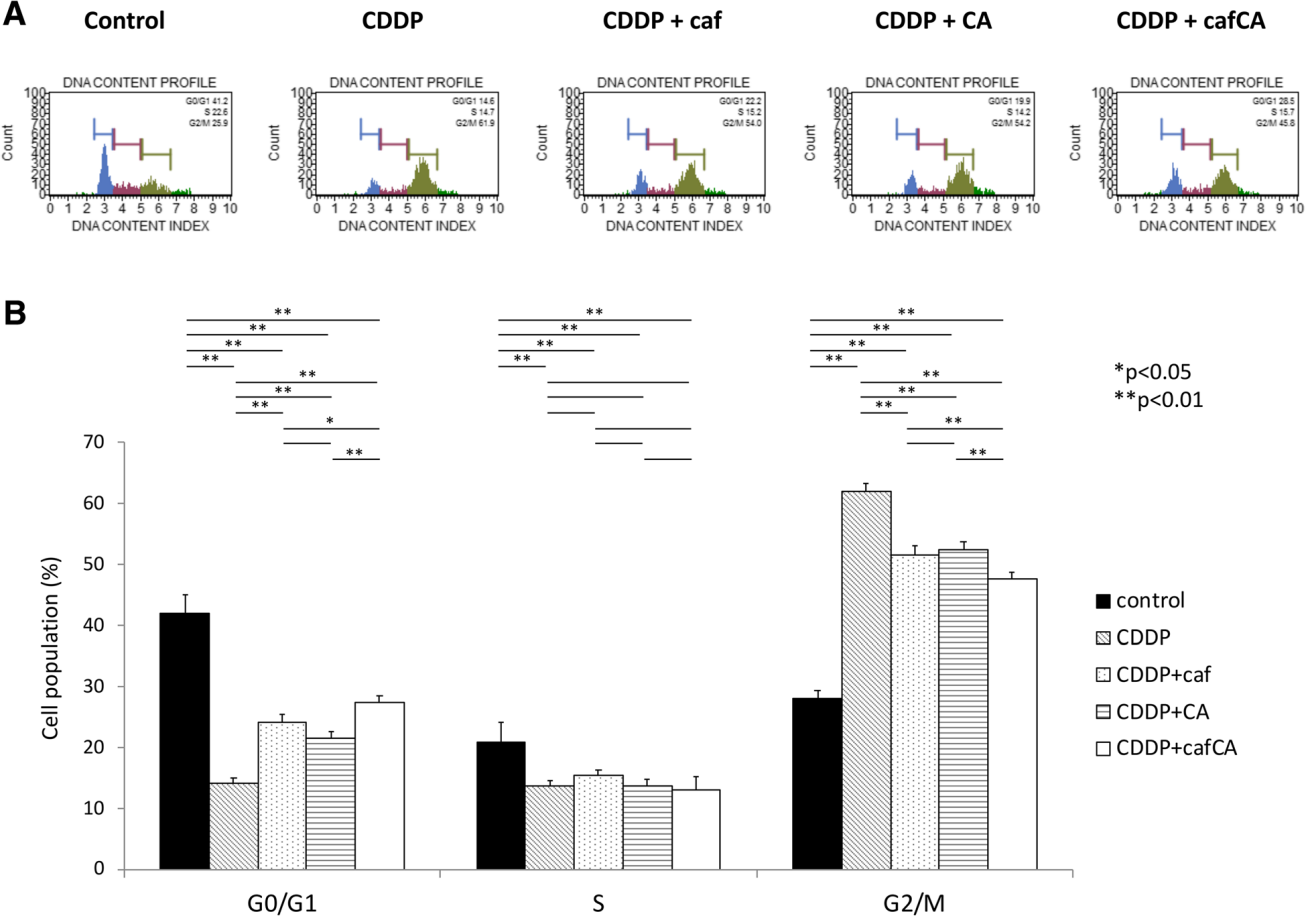
Analysis of cell cycle arrest in treated or untreated human osteosarcoma cells. **a** The DNA content profile. **b** Data are the mean percentages of cell population in five trials with standard deviations

After treatment with cisplatin alone, G2/M fraction was significantly increased, indicating G2/M arrest. After treatment with cisplatin + caffeine, cisplatin + citrate, and cisplatin + caffeine citrate, G2/M fraction was also significantly increased, compared with control group. However, G2/M fraction was significantly decreased, compared with cisplatin alone. These results indicated that the combined treatment with cisplatin proceeded to G0/G1 from G2/M arrest. Moreover, after treatment (all conditions), G0/G1 fraction was significantly decreased, compared with control group, and after treatment with cisplatin + combination drugs, G0/G1 fraction was significantly increased, compared with treatment with cisplatin alone. In S fraction, there was significant differences between control and treatment group, and there was no significant differences between treatment with cisplatin alone and cisplatin + combination drugs. These results indicated that the combination drugs induced G0/G1 arrest. Finally, caffeine citrate had the significantly strong effect compare to the combine with caffeine or citrate (Fig. 5).

### **Effect of cisplatin and combinations on caspase 3/7 activity**

**Fig. 6:**
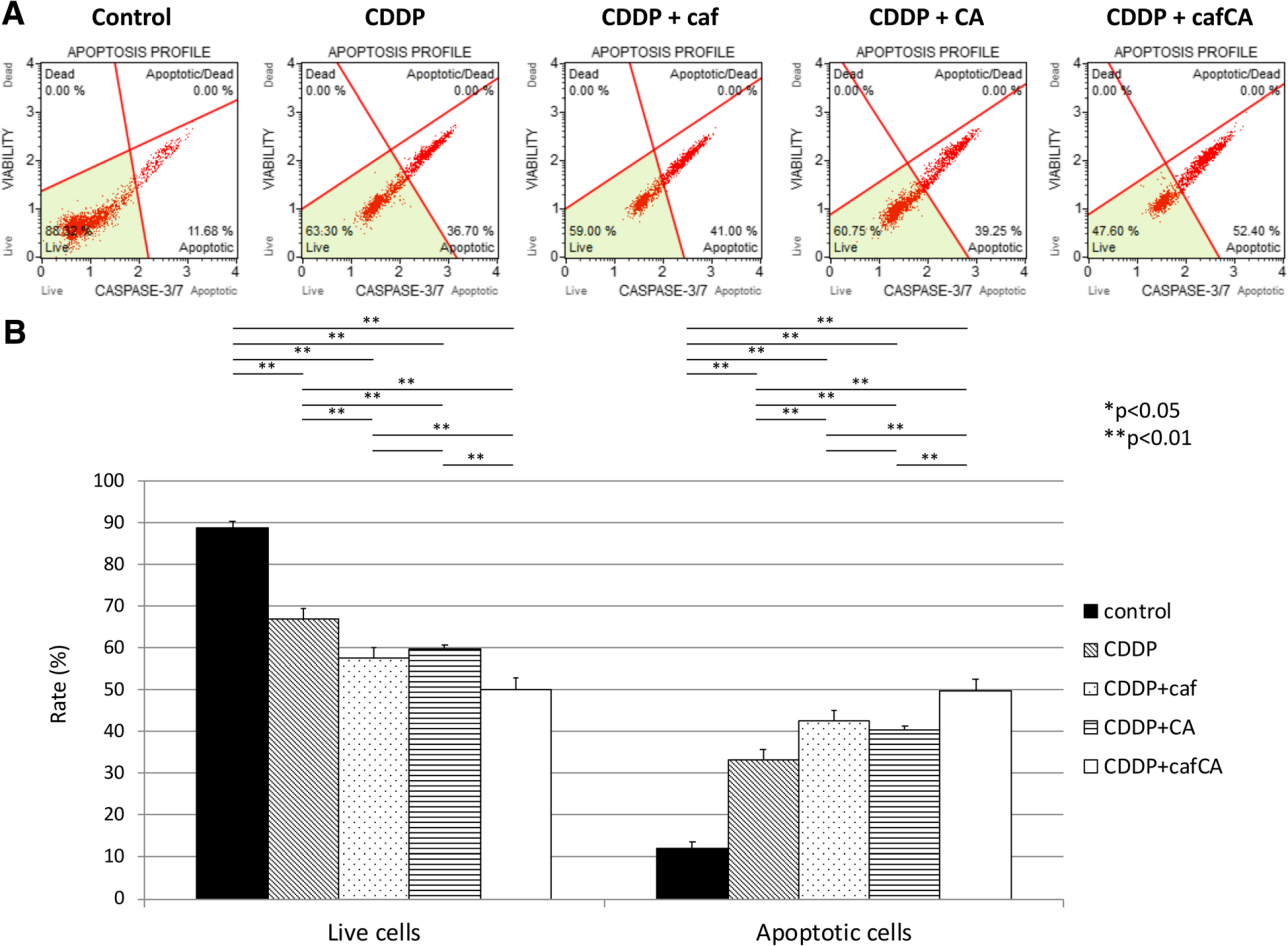
Analysis of caspase 3/7 activation in treated or untreated human osteosarcoma cells. **a** The DNA content profile. **b** Data are the mean caspase 3/7 activation values in five trials with standard deviations

Most of the cells were classified into live and apoptotic cells among live, apoptotic, apoptotic/dead, and dead cells (Fig. 6a). In the rate of live cells, control group was significantly higher than all other treatment group. After treatment with cisplatin + caffeine citrate, the rate of live cells was significantly lower, compared with any other treatment group. After treatment with cisplatin + caffeine and cisplatin + citrate, the rate of live cells was also significantly lower, compared with cisplatin alone. In the rate of apoptotic cells, control group was significantly lower than all other treatment group. After treatment with cisplatin + caffeine citrate, the rate of live cells was significantly higher, compared with any other treatment group. After treatment with cisplatin + caffeine and cisplatin + citrate, the rate of live cells was also significantly lower, compared with cisplatin alone (Fig. 6b).

### **Effect of cisplatin and combinations on mouse tumors**

**Fig. 7:**
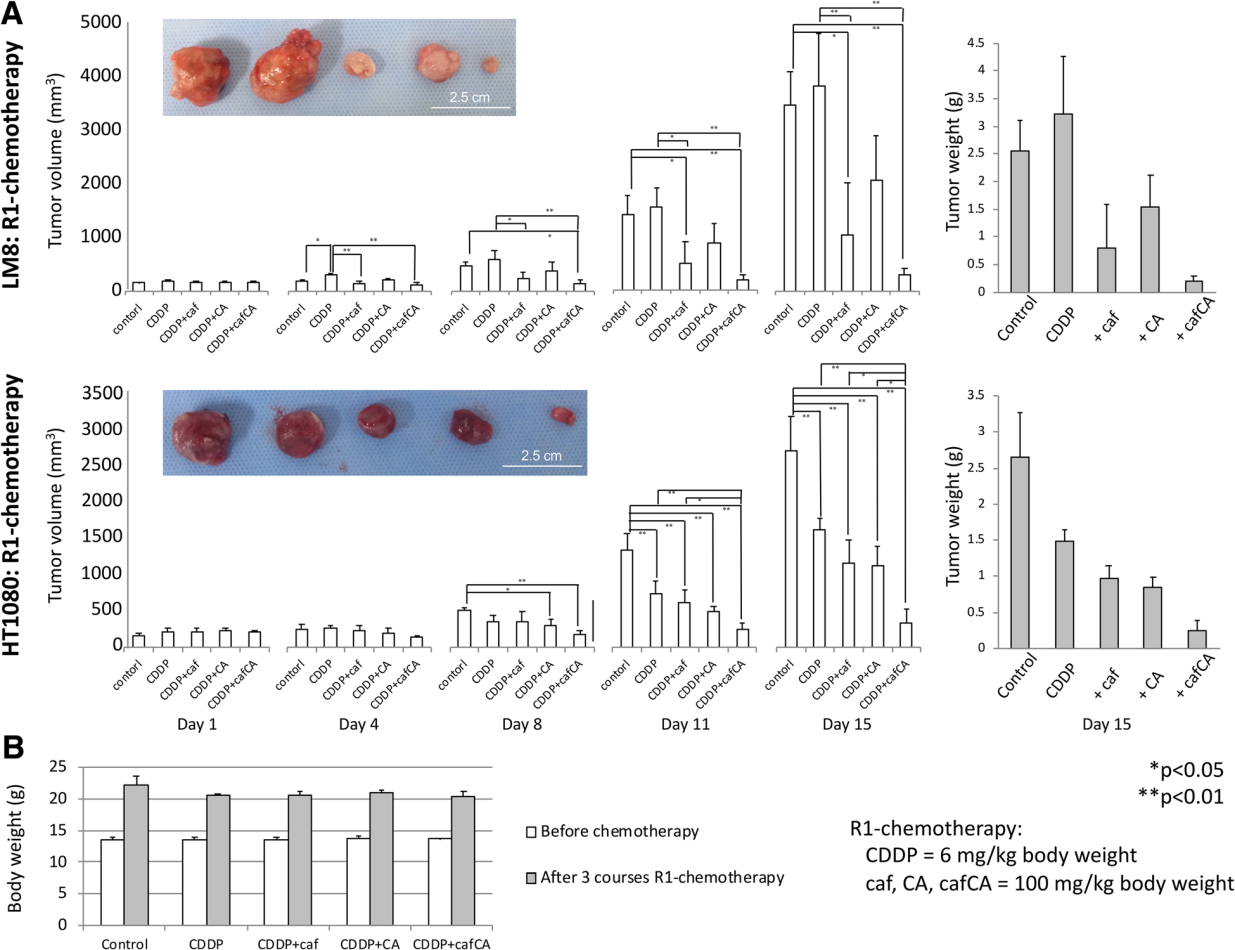
Effects of cisplatin (CDDP), 6 mg/kg of body weight, alone or with caffeine (caf) or citrate (CA) at 100 mg/kg or caffeine citrate (cafCA) at 200 mg/kg (R1-chemotherapy) to treat mice implanted mouse osteosarcoma and human fibrosarcoma. **a** Tumor sizes were measured 2 times a week and the volumes calculated, and mean weights of tumors removed at necropsy. Two panels show an excisional tumor on day 15. From left to right, the tumor treated with saline (control), cisplatin alone, cisplatin with caffeine, cisplatin with citrate, and cisplatin with caffeine citrate. Scale bar = 2.5 cm. **b** Changes in body weight of mice without tumors after 3 courses of chemotherapy (cisplatin [CDDP], 6 mg/kg of body weight, alone or with caffeine [caf] or citrate [CA] at 100 mg/kg or caffeine citrate [cafCA] at 200 mg/kg) to test for adverse effects

2 days after implantation of LM8 mouse osteosarcoma or HT1080 human fibrosarcoma cells, the mice were treated with chemotherapy by intraperitoneal injection 2 times a week for 2 weeks. Cisplatin + caffeine citrate significantly reduced tumor volume and tumor weight in both LM8 and HT1080 by R1 protocol. Especially, the volume of HT1080-tumor treated with cisplatin + caffeine citrate was significantly smaller than with all other treatment (Fig. 7a). The five groups of mice without implanted tumors had no significant differences in body weight after three courses of the R1 chemotherapy regimens (Fig. 7b). In the results of R2 regimen for LM8, the volume and weight of the tumor treated with caffeine + citrate was significantly reduced, compared with control group. There was no significant difference, but compared with other treatments, it was able to suppress the tumor volume and weight (Additional file 1: Figure S1A). In the results of R3 regimen for LM8, the volume and weight of the tumor treated with caffeine + citrate was also significantly reduced, compared with control group. Like R2 regimen, there was no significant difference, but compared with other treatments, it was able to suppress the tumor volume and weight (Additional file 1: Figure S1B).

## Discussion

In this study, we found that chemotherapy with cisplatin + caffeine citrate suppressed osteosarcoma and fibrosarcoma cell proliferation and enhanced apoptosis as compared with cisplatin alone or with the addition of either caffeine or citrate. The effect of caffeine citrate was synergistic, not just additive. We also demonstrated that cisplatin + caffeine citrate had a significantly greater inhibition of tumor growth than the other treatments given to mice with implanted osteosarcomas or fibrosarcomas.

Caffeine inhibits DNA repair induced by cisplatin and increases the anticancer effects of cisplatin [44]. Mechanisms responsible for the influence of caffeine on the anticancer effects of cisplatin have been suggested to involve several proteins, such as ATR kinase, ataxia telangiectasia mutated (ATM) kinase, and p53 unregulated modulator of apoptosis (PUMA) [45, 46]. Caffeine overcomes the cisplatinum-induced S/G2 cell-cycle arrest with subsequent increased apoptosis. Cell-cycle arrest is a survival mechanism in chemotherapy-treated cells, and progression of the cell cycle induced by caffeine resensitizes cancer cells to chemotherapy [47]. In this study, caffeine was stronger, overcame the cisplatinum-induced S/G2 cell-cycle arrest with subsequent increased apoptosis in HOS. Moreover, caffeine induced G0/G1 cell-cycle arrest and this result suggested that caffeine also suppressed cell proliferation.

Citrate leads to an early decrease in the expression of the anti-apoptotic protein Mcl-1, a molecule that plays a key role together with the protein Bcl-xL in the chemoresistance of certain cancers [28, 29], particularly mesothelioma [30]. The addition of citrate to Bcl-xL-expressing cells may lead to increased protein N-alpha-acetylation and sensitization of those cells to apoptosis [31]. The mechanism explaining the sensitization to chemotherapy of citrate-exposed cells remains to be investigated [32]. These findings are mainly from in vitro studies of cells and there are few studies with cell cycle. None have been done using sarcoma cell lines nor are there clinical studies of the effects of adding citrate to chemotherapy. In this study, citric acid also overcame the cisplatinum-induced S/G2 cell-cycle arrest with subsequent increased apoptosis and induced G0/G1 cell-cycle arrest with subsequent suppressed cell proliferation. Cancer cells depend on both glycolysis system and citric acid cycle [48], so we have to continue research on cancer metabolism in the future.

Finally, caffeine citrate had most strong effect as a combination drug than caffeine and citric acid in both overcoming the cisplatinum-induced S/G2 cell-cycle arrest with subsequent increased apoptosis and inducing G0/G1 cell-cycle arrest with subsequent suppressed cell proliferation.

This study also demonstrated an in vitro effect of caffeine citrate potentiating cisplatin. It is intriguing is that this was a synergistic effect, better than that of cisplatin combined with either caffeine or citrate. The separate mechanisms described above by which caffeine and citrate enhance an antitumor effect may have played a role in our findings in this study but that does not account for the synergistic effect of caffeine citrate. In addition to those findings in vitro*,* we have also demonstrated caffeine citrate’s value when added to cisplatin in the in vivo mouse model for both osteosarcoma and fibrosarcoma.

As a limitation, caffeine monotherapy and citrate monotherapy have not been evaluated, and only one kind of cell (HOS) was used in flow cytometry.

## Conclusions

This is the first report demonstrating that caffeine citrate has a significantly greater potentiating effect on cisplatin than adding either caffeine or citric acid. Further investigation is of course required before this can be tested clinically, but the combination of cisplatin with caffeine citrate is a novel treatment that might hold promise for improving the outcome of osteosarcoma and fibrosarcoma, which up till now has generally not responded well to chemotherapy. It would also be worthwhile evaluating whether caffeine citrate might potentiate chemotherapy for other types of cancer.

## Additional files

Additional file 1: **Figure S1.** The results of R2 and R3 regimen for LM8. Effects of cisplatin (CDDP), 6 mg/kg of body weight, alone or with caffeine (caf) or citrate (CA) at 50 mg/kg or caffeine citrate (cafCA) at 100 mg/kg (R2-chemotherapy) and cisplatin (CDDP), 3 mg/kg of body weight, alone or with caffeine (caf) or citrate (CA) at 100 mg/kg or caffeine citrate (cafCA) at 200 mg/kg (R3-chemotherapy) to treat mice implanted mouse osteosarcoma. Tumor sizes were measured 2 times a week and the volumes calculated, and mean weights of the tumors removed at necropsy. (PDF 466 kb)

Additional file 2: **Table S1.** The information on quantitative changes. (XLSX 19 kb)

## Data Availability

The datasets generated and analyzed during the current study are not publicly available because we will continue to use them for future research but are available from the corresponding author on reasonable request. The information on quantitative changes is included in Additional file 2: Table S1

## Abbreviations

CA: Citrate
caf: Caffeine
cafCA: Caffeine citrate
CDDP: Cisplatin
CI: Combination index
EdU: 5-ethynyl-2-deoxyuridine
IC_50_: Half-maximal inhibitory concentration
TMRE: Tetramethylrhodamine methyl ester
WST-8: 4-[3-(4-iodophenyl)-2-(4-nitrophenyl)-2H-5-tetrazolio]-1,3-benzene disulfonate
