# Supplementary material for: Caffeine citrate enhanced cisplatin antitumor effects in osteosarcoma and fibrosarcoma in vitro and in vivo
Source: BMC Cancer. 2019 Jul 15;19:689. doi: 10.1186/s12885-019-5891-y (PMC6631922; doi:10.1186/s12885-019-5891-y)
Supplement: Supplementary file 1 — Figure S1. The results of R2 and R3 regimen for LM8. Effects of cisplatin (CDDP), 6 mg/kg of body weight, alone or with caffeine (caf) or citrate (CA) at 50 mg/kg or caffeine citrate (cafCA) at 100 mg/kg (R2-chemotherapy) and cisplatin (CDDP), 3 mg/kg of body weight, alone or with caffeine (caf) or citrate (CA) at 100 mg/kg or caffeine citrate (cafCA) at 200 mg/kg (R3-chemotherapy) to treat mice implanted mouse osteosarcoma. Tumor sizes were measured 2 times a week and the volumes calculated, and mean weights of the tumors removed at necropsy. (PDF 466 kb) [file 12885_2019_5891_MOESM1_ESM.pdf]

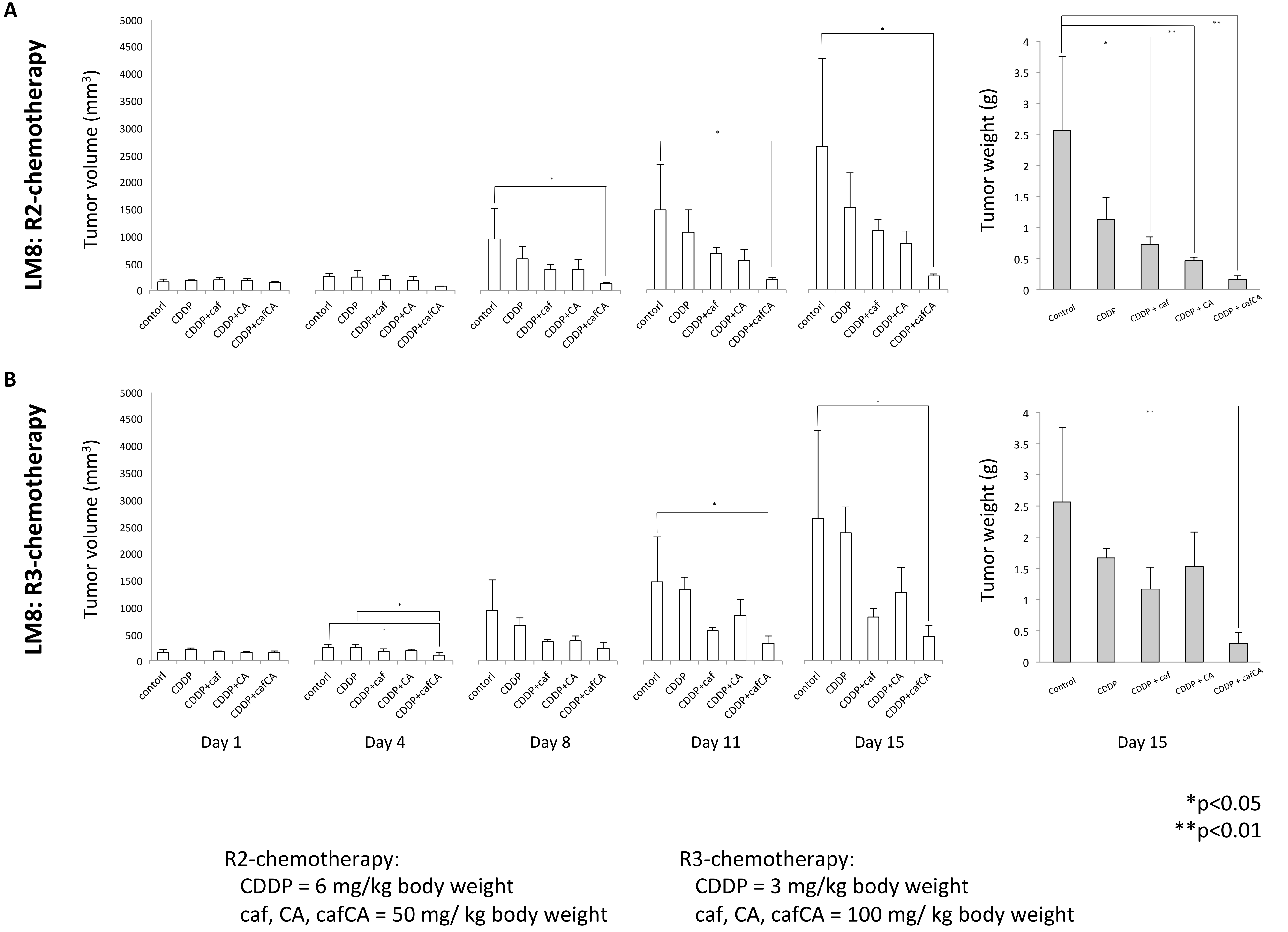

A
LM8: R2-chemotherapy
Tumor volume (mm³)
Tumor weight (g)
contorl
CDDP
CDDP+caf
CDDP+CA
CDDP+cafCA
Control
CDDP + caf
CDDP + CA
CDDP + cafCA
B
LM8: R3-chemotherapy
Day 1
Day 4
Day 8
Day 11
Day 15
*p<0.05
**p<0.01
R2-chemotherapy:
CDDP = 6 mg/kg body weight
caf, CA, cafCA = 50 mg/ kg body weight
R3-chemotherapy:
CDDP = 3 mg/kg body weight
caf, CA, cafCA = 100 mg/ kg body weight
